# Simultaneous Bilateral Anterior Glenohumeral Fracture Dislocation: A Case Report

**DOI:** 10.7759/cureus.24199

**Published:** 2022-04-17

**Authors:** Hardik L Patel, Suraj J Babar, Vijayanand Balasubramanian, Sabari Vaasan Lakshmi Kanthan, Adnan Ahmed

**Affiliations:** 1 Orthopedic Surgery, Sri Ramaswamy Memorial (SRM) Institute of Science and Technology, Chennai, IND; 2 Orthopedics, Sri Ramaswamy Memorial (SRM) Medical College Hospital and Research Centre, Chengalpattu, IND

**Keywords:** closed reduction of shoulder, deltopectoral, simultaneous bilateral shoulder dislocation, shoulder dislocation, glenohumeral joint dislocation

## Abstract

Simultaneous anterior glenohumeral dislocations are rare in occurrence and difficult to diagnose and treat. Here, we present a case of a 33-year-old male with simultaneous anterior glenohumeral fracture dislocation after an episode of seizure. Closed reduction of both the shoulders was performed. Displaced greater tuberosity fracture fixation was done through deltoid splitting approach using cannulated cancellous screws. Fracture union was achieved at three months of follow-up with a good functional outcome. Early diagnosis and reduction provide a good functional outcome.

## Introduction

The most common type of joint dislocation in the human body is the glenohumeral joint with a 1.7% incidence rate, often seen in contact and/or overhead sports [1,2]. Simultaneous anterior dislocation occurs with a lesser frequency than simultaneous posterior dislocation and most often occurs due to trauma [3,4]. These injuries can be missed easily if the examination is not thorough. A detailed clinical and radiological evaluation is essential to rule out associated fracture or neurovascular injury [5]. The reported causes of injury-causing simultaneous anterior fracture-dislocation are fall, electrocution, and weight lifting [6]. Isolated displaced greater tuberosity fractures occur in less than 2% of proximal humeral fractures and are usually noted with anterior shoulder dislocations [7]. We are reporting a case of simultaneous anterior glenohumeral fracture dislocation following a seizure, treated surgically.

## Case presentation

**Figure 1:**
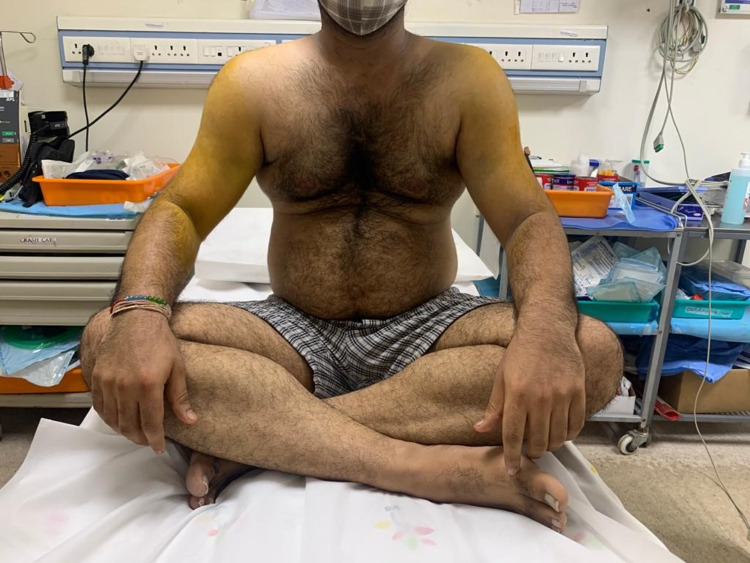
Clinical image demonstrating squaring of shoulders and abduction

**Figure 2:**
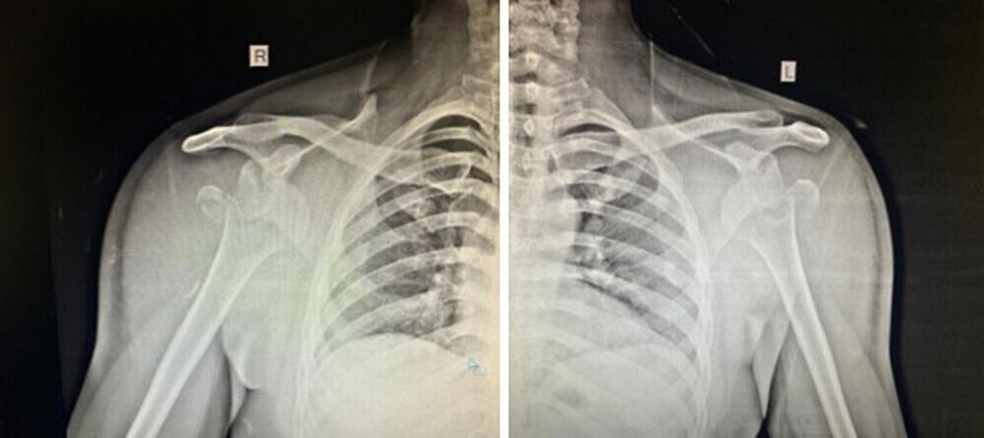
Pre-reduction radiographs showing bilateral shoulder dislocation with displaced greater tuberosity fractures

A 33-year-old male fell down following an episode of seizure three days back and presented with difficulty in lifting his shoulders. He had no previous history of shoulder dislocation and he was not taking any anti-epileptic medication. He was diagnosed with bilateral anterior shoulder dislocations with symmetrical greater tuberosity fractures by radiographs. Closed reduction was attempted at an outside hospital and failed. On examination, both the shoulders were fixed in abduction with squaring of shoulders without evidence of peripheral motor, sensory, or vascular deficit (Figure 1). Radiographs were performed showing bilateral glenohumeral dislocation with associated displaced fractures of greater tuberosity (Figure 2).

**Figure 3:**
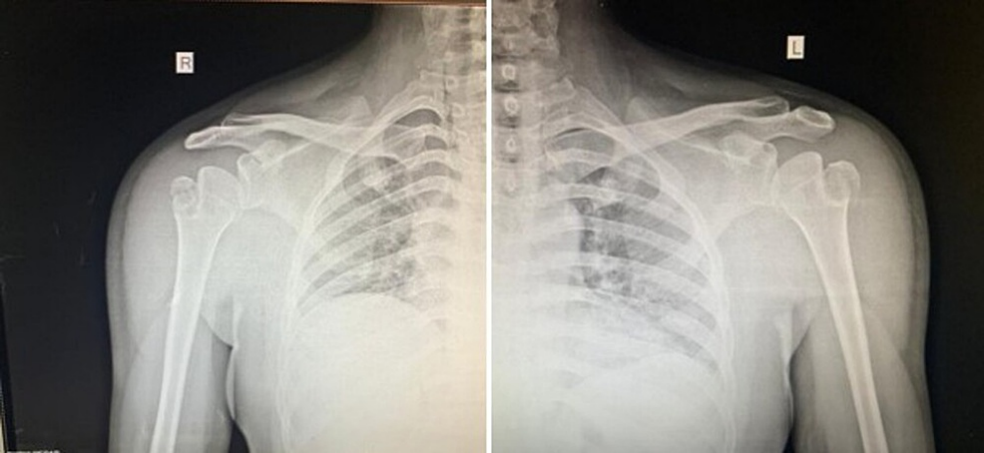
Post-reduction radiographs of bilateral shoulders

**Figure 4:**
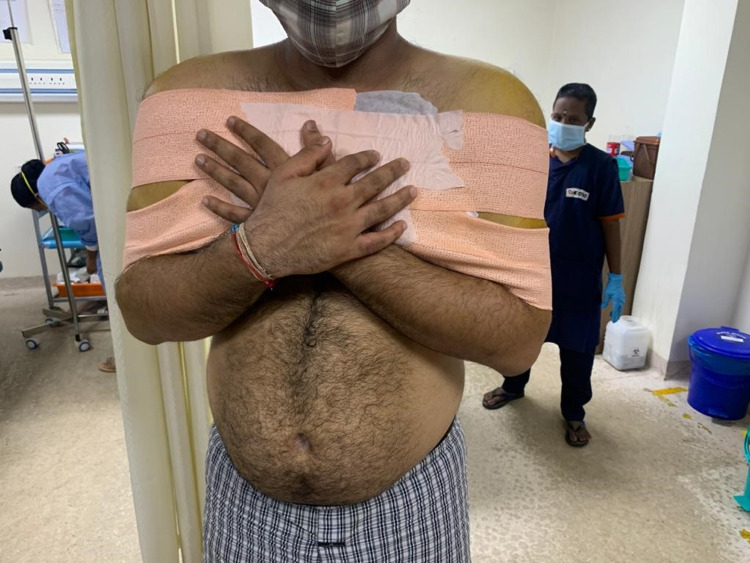
Strapping of both the shoulders

Closed reduction of both the shoulders was done by traction-countertraction technique under intravenous sedation using propofol (Figure 3). The technique involves the patient lying supine and countertraction is provided by a sheet wrapped around the upper thorax of the patient. The surgeon can then stand on the side of the dislocated shoulder holding the forearm of the patient with the elbow flexed to 90 degrees. Against the assistant’s countertraction, slow and steady traction is applied to the patient’s arm to distract the humeral head away from the glenoid and disengage it from the glenoid rim. At reduction, the affected arm is usually lengthened and relaxed with an audible clunk. Both the arms were strapped to the chest after using adequate padding (Figure 4).

**Figure 5:**
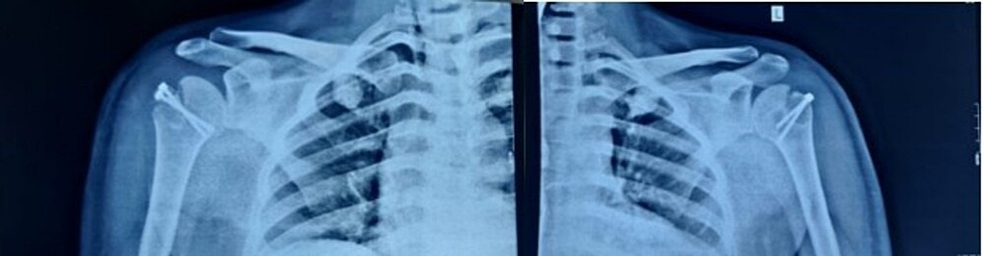
Immediate post-operative radiograph of bilateral shoulder

CT scan of both shoulders showed displaced greater tuberosity fracture. MRI of both shoulders was performed which showed grade II tear in supraspinatus. The patient was taken up for open reduction and cannulated cancellous screw fixation for displaced greater tuberosity fracture of bilateral shoulders. Surgery was done under general anesthesia. Using deltoid splitting approach the fracture fragments were found to be large and non-comminuted and were reduced and fixed with two 4.5 mm cannulated cancellous screws (Figure 5). 

**Figure 6:**
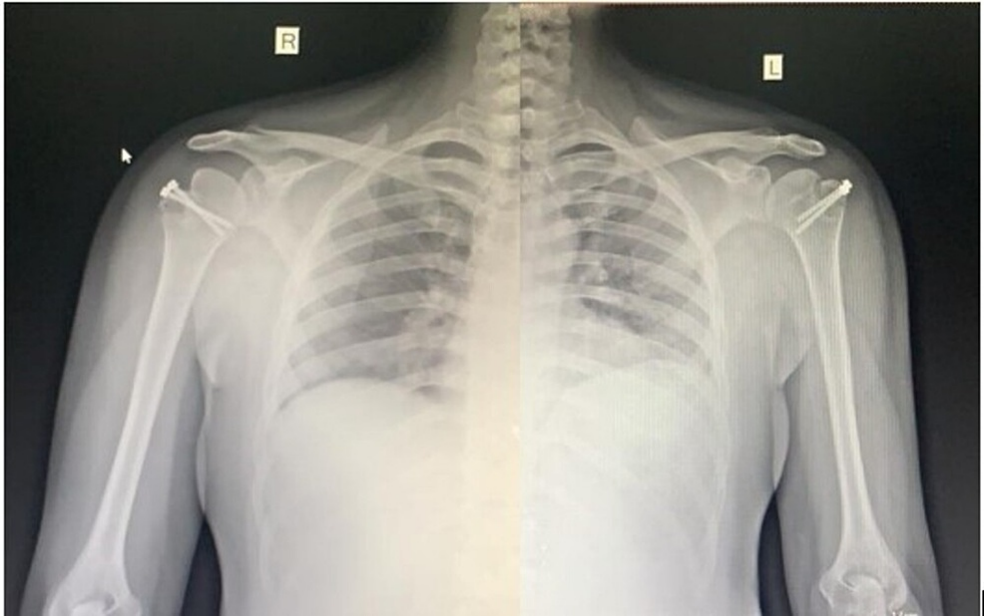
Three months post-operative radiograph

Partial tear of supraspinatus was noted in both shoulders, hence repair was not done. Both the shoulders were immobilized for two weeks till suture removal, then pendulum exercises and range of motion (ROM) exercises started. Both greater tuberosity fractures united at three months of follow-up (Figure 6, Table 1). 

**Table 1:** Range of motion at three months of follow-up

	Right (degrees)	Left (degrees)
External rotation	80	90
Internal rotation	60	65
Forward flexion	180	180
Abduction	180	180

**Figure 7:**
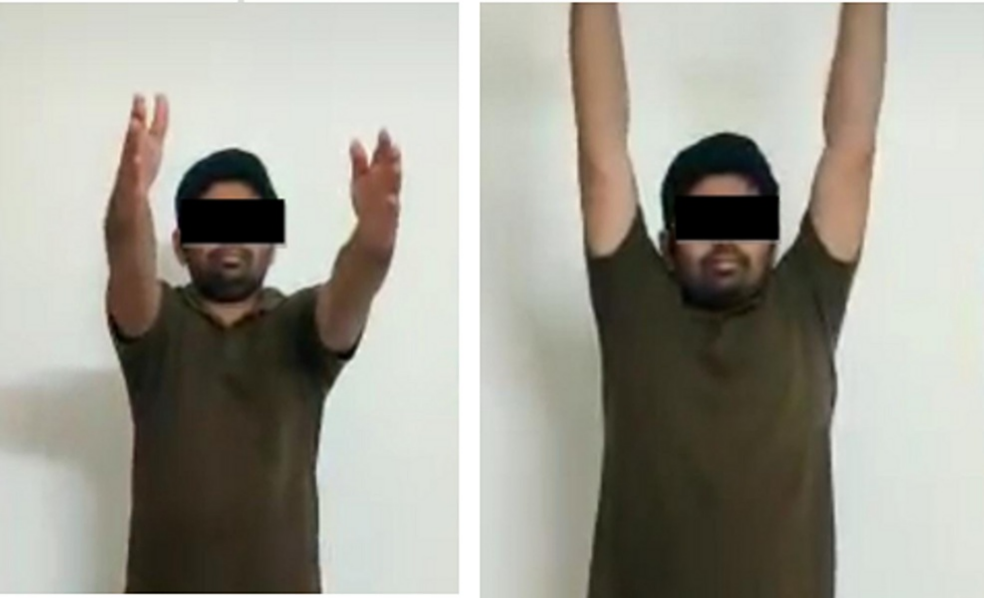
Clinical pictures of functional outcome

Figure 7 shows the clinical outcome at a three-month follow-up. Motor strength of bilateral rotator cuff muscles was 5/5 according to the medical research council (MRC) grading.

## Discussion

Mynter described bilateral shoulder dislocation for the first time in 1902 in a patient with excessive muscular contractions as a result of camphor overdose [8]. The main cause of bilateral anterior shoulder dislocation is trauma to the shoulders in the abduction, extension, and external rota­tion position [5]. The injury mechanics causing bilateral dislocation after trauma does not differ from that of isolated shoulder joint dislocation. But the force which leads to dislocation occurs at the same time in a similar fashion on both the shoulders [9]. Traction in forward flexion of the shoulder is the other mechanism of injury.

In literature, reported cases are due to sport-related injuries including weight-lifting exercise, back­stroke swimming, and horse rid­ing. Other than traumatic causes, bilateral anterior shoulder dislocation can occur after hypoglycemic convul­sion or epileptic seizure. Bilateral anterior dislocation after seizure may be due to the trauma of the shoul­ders striking the floor after the collapse [5,10]. Most of the patients remain delirious or in an unconscious state in the postictal period, making them prone to secondary insult and causing a bilateral dislocation due to loss of inhibition and reflex protective mechanisms [9]. The posterior dis­locations are common after seizures because the con­traction of the relatively weak external rotators and posterior fibers of deltoid are overcome by the more powerful internal rotator (subscapularis). The succeeding adduction and internal rotation usually cause the humeral head to dislocate posteriorly [1,5]. 

In the literature on bilateral dislocations, 80% of cases were traumatic in origin and 20% of cases were non-traumatic; either due to seizures or electric shock. No patients were found below 20 years of age, 37% of the patients were between 20 and 49 years, 32% of the patients were between 50 and 69 years, and 31% of the patients were 70 years and above. Ten percent of patients out of all bilateral shoulder dislocations were evaluated with computed tomography (CT) and 15% were evaluated with magnetic resonance imaging (MRI). The rarity of this conundrum and the false symmetry of the shoulder girdle during examination - compared to the typical asymmetry in the unilateral dislocation delays the diagnosis. Dunlop estimated that > 10% of all bilateral shoulder dislocations had delay in diagnosis [5].

According to available literature, 43% of patients had simple dislocation and 57% of patients had fracture dislocation; out of which, greater tuberosity fracture was most commonly associated (50%) followed by three-part and four-part fractures of proximal humerus (~17% each). Ten percent of patients had associated brachial plexus injury (radial nerve; posterior and inferior cord) and 15% of patients had rotator cuff injuries (supraspinatus). Ninety percent of patients presented to hospital on the same day of trauma and 10% presented after two weeks of trauma.

Bilateral anterior shoulder dislocations in patients > 40 years are frequently accompanied by proximal humeral fractures or soft-tissue injuries (e.g., rotator cuff tears or brachial plexus injuries). While the fractures are detected on x-rays, the soft-tissue pathologies are often missed and have a significant negative effect on the outcome [11]. Eighty-five percent of patients were treated with closed reduction. Fifteen percent of patients were treated with open reduction and some form of fixation who presented late with fracture dislocation. Good results with bilateral anterior dislocation depend largely on early precise diagnosis. Late presentation and diagnostic difficulties were documented in the literature and this delay necessitates a large number of open reductions, with the correspondingly poorer result. Fifty percent of patients who presented late required open reductions. Only early and prompt treatment will ensure a good functional outcome [12].

## Conclusions

Simultaneous anterior glenohumeral dislocation with associated fracture of the greater tuberosity bilaterally is an extremely rare occurrence. A thorough physical examination helps in early diagnosis. Soft tissue injuries accompany them frequently. Prompt reduction and stabilization are necessary for good functional outcomes.
